# Hippocampal subfield thickness and shape analysis in examining the impact of TDP‐43 in primary age‐related tauopathy

**DOI:** 10.1002/alz.71267

**Published:** 2026-03-08

**Authors:** Hossam Youssef, Rodolfo G. Gatto, Ronald C. Petersen, R. Ross Reichard, Clifford R. Jack, Jennifer L. Whitwell, Keith A. Josephs

**Affiliations:** ^1^ Department of Neurology Mayo Clinic Rochester Minnesota USA; ^2^ Department of Laboratory Medicine and Pathology, Mayo Clinic Rochester Minnesota USA; ^3^ Department of Radiology Mayo Clinic Rochester Minnesota USA

**Keywords:** geometry‐based analysis, hippocampal deformation, hippocampal shape analysis, hippocampal subfields, PART, surface‐based analysis, TDP‐43

## Abstract

**INTRODUCTION:**

Hippocampal subfields are vulnerable to the transactive response DNA‐binding protein of 43 kDa (TDP‐43) and tau in primary age‐related tauopathy (PART). Geometry‐based morphometric analysis can improve the detection of structural changes in the hippocampus.

**METHODS:**

Forty‐seven cases of autopsy‐confirmed PART without TDP‐43 and 19 cases of PART with TDP‐43 underwent *antemortem* magnetic resonance imaging (MRI) hippocampal segmentation and shape analysis. A separate cohort of 16 younger healthy individuals (YHIs) was included as a reference group. Hippocampal shape was analyzed in Python with non‐parametric cluster permutation testing.

**RESULTS:**

PART(TDP+) group exhibited thinner left subiculum and CA1, with curvature deformations in left CA1 and CA2/3, relative to PART(TDP–). Both PART groups showed right‐sided predominance of thinning in presubiculum and subiculum, as well as curvature deformations in right presubiculum, subiculum, and CA1 when compared to YHIs.

**DISCUSSION:**

Geometry‐based analysis showed different patterns of hippocampal thinning and curvature associated with PART(TDP+) compared to PART(TDP–).

## Highlights

- Geometry‐based unfolding algorithm applied to hippocampal subfield analysis.
- Primary age‐related tauopathy (PART) cases show reduced thickness in the presubiculum and subiculum.
- Right hippocampal subfields show stronger thinning and curvature changes.
- Transactive response DNA‐binding protein positive (TDP+) cases reveal thinning in the left subiculum and CA1 versus TDP−.
- Curvature deformations in CA1 and CA2/3 with TDP of 43 kDa (TDP‐43) presence.

## 1 BACKGROUND

Primary age‐related tauopathy (PART) is a neuropathology observed in the brains of the elderly.^1^ It is characterized by the presence of neurofibrillary tangles (NFTs) [Braak neurofibrillary tangle (NFT) stage I–IV)] in the absence of significant amyloid‐β (Aβ) plaques (Thal Aβ stage 0–2).^1^, ^2^ PART can be considered as distinct from Alzheimer's disease, where Aβ deposition (Thal 3–5) and NFT deposition (Braak V–VI) are more widespread. In PART, the hippocampus, which is essential for memory formation, is particularly vulnerable and shows characteristic morphological changes in response to pathological aggregates.^3^, ^4^, ^5^, ^6^ One prior study reported finding an association between the Braak NFT stage and atrophy of the left head of the hippocampus in PART.^7^

The transactive response DNA‐binding protein of 43 kDa (TDP‐43) has been linked to neurodegeneration and neurodegenerative diseases.^3^, ^8^, ^9^, ^10^, ^11^ TDP‐43 has been found to co‐deposit in cases of PART, with the frequency of TDP‐43 increasing with age.^12^ Some researchers have advocated for labeling TDP‐43 in the elderly as limbic predominant age‐related TDP‐43 encephalopathy–neuropathologic changes (LATE‐NC).^13^ In PART, TDP‐43 has been shown to be associated with greater atrophy in the amygdala, lateral temporal cortex, and hippocampus,^14^ particularly the CA1 and subicular subfields of the hippocampal body.^15^

In addition to conventional techniques assessing hippocampal volume, geometry‐based morphometric analysis techniques have been developed to allow the assessment of hippocampal shape.^16^, ^17^, ^18^ These methods have demonstrated superior performance in detecting disease‐related changes compared to traditional approaches.^17^, ^18^ Recent research showed that some hippocampal subfields exhibit volumetric changes in association with TDP‐43 in PART.^15^ Other cohorts, excluding PART, have indicated vulnerabilities in the shape of hippocampus with TDP‐43.^19^, ^20^ TDP‐43 is associated with temporal lobe changes, inward deformation of bilateral CA1 and subiculum, and head of the left hippocampus.^3^, ^19^, ^20^, ^21^, ^22^ Few surface‐based deformation studies identified the subiculum and CA1 as the hippocampal subfields most strongly associated with tau pathology.^20^, ^23^ However, it is unclear whether TDP‐43 and tau exert distinct influences on hippocampal thickness and shape curvature in cases of PART. Additionally, it is unclear whether all subfields are uniformly susceptible to TDP‐43 and tau proteinopathies, or if only certain subfields are affected.

The aim of this study was to determine whether TDP‐43 is associated with volume and shape deformation across the hippocampal subfields in PART. To address this aim, we implemented a geometry‐based algorithm that generates a geometric representation of the internal structure of the hippocampus.^17^, ^18^ This algorithm utilizes hippocampal unfolding to establish an intrinsic anatomical coordinate system, facilitating point‐wise analysis of morphometric alterations of the hippocampal subfields.^17^, ^18^ We hypothesize that TDP‐43 and tau lead to structural thinning of the hippocampal subfields, but with distinct curvature alterations in PART.

## 2 METHODS

### 2.1 Study population

We have described our selection criteria in a recent study.^15^ To summarize, cases that met the criteria for PART^1^ were identified from a cohort of 1720 autopsy‐confirmed cases from the Mayo Clinic Alzheimer's Disease Research Center (ADRC), the Mayo Clinic Study of Aging (MCSA), and the Neurodegenerative Research Group (NRG).

### 2.2 PART diagnostic criteria

PART was diagnosed according to the consensus criteria.^1^ Inclusion required: (1) neurofibrillary tangles (NFTs) in medial temporal lobe structures, (2) Braak NFT stage I–IV, (3) Thal amyloid‐β (Aβ) phase 0–2, and (4) absence of significant neuritic plaques. Cases were classified as “Definite PART” (Thal phase 0) or “Possible PART” (Thal phase 1–2). The National Institute on Aging and Alzheimer's Association (NIA‐AA) criteria^24^ were utilized for a standardized neuropathological examination, which included tissue sampling and a semi‐quantitative assessment of Alzheimer's disease pathology. Braak NFT stage and Thal Aβ stage were assigned to the cases in accordance with NIA‐AA recommendations.^25^, ^26^, ^27^ NFTs and Aβ semi‐quantitation and staging were conducted through the visual inspection of an expert neuropathologist.

### 2.3 TDP‐43 pathological assessment

All neuropathological assessments, including TDP‐43 immunohistochemistry, were conducted on the left hemisphere, as described in previous studies.^9^, ^15^, ^28^, ^29^, ^30^ The right hemisphere was frozen at the time of brain harvesting. Subsequently, blank slides were immunostained to identify TDP‐43 using a conformation‐specific antibody that targets the C‐terminal fragment of TDP‐43 (MC2085; a generous contribution from Leonard Petrucelli).^31^ Immunostaining was conducted utilizing a DAKO‐Autostainer machine, employing 3, 3′‐diaminobenzidine as the chromogenic indicator, followed by the application of a light Hematoxylin stain. Stained slides of the amygdala and hippocampus were meticulously examined by a neuropathologist (D.W.D.) and a neuroscientist (K.A.J.) to evaluate the presence of TDP‐43 immunoreactive inclusions and to characterize the type of TDP‐43.^28^ Our focus on these two regions is based on prior research conducted by us and others, which has demonstrated that these areas are among the first to be impacted by TDP‐43 in the context of PART.^29^, ^32^ Cases were examined at a magnification of 200× and were classified as positive if any TDP‐43 immunoreactive inclusions were identified, including neuronal cytoplasmic inclusions, dystrophic neurites, neuronal intranuclear inclusions, fine neurites in the CA‐1 region of the hippocampus, NFT‐associated TDP‐43 (TATs), as well as perivascular and granular inclusions. Conversely, if no TDP‐43 inclusions were detected in either the amygdala or hippocampus, the case was categorized as TDP‐negative. TDP‐43 stage was also determined as previously described on a scale from stage 1 (amygdala only) to stage 6 (extending to frontal cortex or basal ganglia).^9^, ^29^

### 2.4 Sample selection and exclusion

Initially, 115 cases met PART criteria. We excluded all 1.5T MRI scans and retained only 3T scans, yielding 73 cases with adequate imaging. The cases underwent at least one *antemortem* GE 3T MRI brain scan and *post‐mortem* brain autopsy between January 1, 1999 and December 31, 2022.

Alongside the PART cases, we identified 16 younger healthy individuals (YHIs) from the MCSA who underwent 3T MRI, were negative for Aβ deposition on positron emission tomography (PET) and were 50 years of age or younger at the time of the scan. The YHIs were selected as a reference group to provide an anatomical reference standard for normal hippocampal morphometry. It is not biologically feasible to select an age‐matched control group without PART pathology, as nearly all individuals who die in the eighth to ninth decades harbor at least Braak stage I–II tau pathology. We emphasize that comparisons between PART groups and the YHIs are presented as contextual reference for the magnitude of hippocampal abnormalities rather than age‐adjusted inferential comparisons. Our primary inferential analyses focus on the age‐matched comparison between PART (TDP+) and PART (TDP–) groups.

**RESEARCH IN CONTEXT**

1. **Systematic review**: The authors reviewed the previous literature via traditional sources and search engines. Literature suggests that hippocampal subfields are vulnerable to transactive response DNA‐binding protein of 43 kDa (TDP‐43) in conjunction with primary age‐related tauopathy (PART). Previous studies noted hippocampal subfields’ volumetric changes associated with TDP‐43, but it remained unclear whether TDP‐43 exert distinct influences on the thickness and shape curvature in PART. Geometry‐based morphometric analysis, which has demonstrated superior performance in detecting disease‐related changes compared to traditional volume approaches, was implemented to address this.
2. **Interpretation**: Geometry‐based shape analysis revealed distinct patterns of morphometric deformation. PART (TDP‐) cases were associated with more extensive right hippocampal thinning and curvature deformations, particularly in the presubiculum and subiculum. In contrast, PART (TDP+) cases involved thinning and curvature deformation primarily in the left CA1, followed by thinning of the left subiculum and curvature deformation in the CA2/3 subfields. These findings indicate potential distinct lateralization in the effects of TDP‐43 and PART.
3. **Future directions**: Further investigation is necessary, specifically utilizing TDP‐43 immunohistochemical staining on the right hemisphere to confirm that the observed lateralized effects are not attributable only to the left hemisphere pathological analysis. Corroborative ex‐vivo histological studies are needed to validate the histological foundations of the proposed geometry measurements. Future research should also explore surface curvature as a potentially more sensitive early marker for neurodegeneration than thickness or volume changes in PART.

### 2.5 MRI acquisition and quality control

All MRI scans were conducted by 3T GE Medical Systems scanners (SIGNA platform) at Mayo Clinic Rochester, following a standardized magnetization‐prepared rapid gradient echo (MPRAGE) protocol. The evaluation of all MRI scans was performed by the Mayo Clinic ADRC imaging core, employing a 4‐point quality grading scale. In this scale, grades 1–3 denote acceptable quality, while grade 4 signifies scans that are deemed unusable. Detailed acquisition parameters and quality gradient scale are provided in Tables S1 and S2, respectively.

### 2.6 MRI hippocampal segmentation and shape analysis

If cases had more than one *antemortem* MRI, the last MRI closest to death was chosen for analysis. The MRI scans were processed using the recon‐all FreeSurfer 8.0.0 stable release, followed by the segmentHA_T1.sh toolbox to achieve segmentation of the hippocampal subfields.^33^ Subsequently, FreeSurfer hippocampal subfield segmentations were processed using the HIPSTA (Hippocampal Shape and Thickness Analysis) algorithm, an automated geometry‐based method that produces a parametric shape representation of the hippocampus.^17^, ^18^

### 2.7 HIPSTA algorithm pipeline

Hippocampal thickness and curvature were computed using the HIPSTA algorithm, a geometry‐based approach that creates a parametric shape representation of the hippocampus (https://github.com/Deep‐MI/hipsta), with surface mesh processing and Laplace equation solutions implemented using the LaPy library (https://github.com/Deep‐MI/LaPy). A 3D tetrahedral mesh model was generated from FreeSurfer's hippocampal subfield segmentation using the marching cubes algorithm, with the surface mesh undergoing mild Laplacian smoothing to improve triangle quality and the interior filled with volumetric tetrahedral elements using GMSH software (https://gmsh.info/). Details regarding hippocampal boundary definition, localization techniques, mapping, local thickness analysis, and Gaussian curvature analyses are outlined in the Supporting Information Methods Section.

A total of 66 out of the 73 cases, along with 14 out of the 16 YHIs, achieved successful bilateral hippocampal segmentation and shape analysis. It is important to note that seven cases and two YHIs were excluded due to HIPSTA processing failure and topological defects in either the left or right hippocampal surface. This resulted in three groups: PART (TDP–) (*n* = 47), PART (TDP+) (*n* = 19), and YHIs (*n* = 14), which were subsequently entered into the statistical analysis.

### 2.8 Statistical analysis

Continuous demographic data and hippocampal volumes were analyzed across the three groups using the Kruskal–Wallis test (Python v3.13.3). Additionally, the Mann‐Whitney U test was used to compare data between the PART TDP‐43 (–) and (+) subgroups. Categorical variables were assessed between PART subgroups via the chi‐square test. The *p*‐value is set at <0.05.

To address potential confounding by differential distributions of Thal Aβ phase, CERAD neuritic plaque score, and Braak NFT stage between PART subgroups, we performed three complementary sensitivity analyses. First, we conducted a Thal 0‐restricted analysis comparing PART(TDP+) versus PART(TDP−) within only definite PART cases (Thal phase 0), thereby eliminating any Aβ confounding. Second, we performed a CERAD 0‐restricted analysis comparing PART subgroups within cases lacking neuritic plaques, which additionally resulted in a Braak I‐homogeneous subset. Third, we performed analysis of covariance (ANCOVA) for volumetric comparisons between PART groups adjusted for Thal phase, Braak stage, age, and sex. Partial *η* ^2^ (eta‐squared) effect sizes were calculated for ANCOVA models, with interpretation: small (0.01), medium (0.06), large (0.14).

Hemispheric asymmetry was formally assessed using: (1) laterality index quantifying directional asymmetry, (2) asymmetry index measuring absolute percentage difference between hemispheres, and (3) mixed‐effects models testing Group × Hemisphere interactions.

To examine the relationship between hippocampal morphometry and pathology burden, Spearman rank correlation coefficients were computed between mean thickness, mean Gaussian curvature, and neuropathological staging variables (Braak NFT stage, Thal amyloid phase) across all PART cases. Subject‐level morphometric values were calculated by averaging thickness and curvature measurements across all grid vertices per hemisphere. Correlations were performed separately for all PART cases combined, PART(TDP−) and PART(TDP+) cases. Additionally. To assess whether thickness and curvature represent independent morphometric features, Pearson correlations between thickness and Gaussian curvature were computed within subjects.

The point‐wise cluster‐based permutation test for hippocampal curvature and thickness analysis was conducted in Python 3.13.3. Raw measurements were processed using Pandas^34^ to organize surface curvature data and thickness measurements. Statistical analysis employed MNE‐Python's^35^ non‐parametric cluster‐based permutation testing with the following parameters: 5000 permutations for stability, cluster mass statistic for cluster‐level inference, two‐sample *t*‐tests per vertex, spatial adjacency on the 41 × 21 grid, family‐wise error rate (FWER) controlled via maximum statistic permutation distribution, significance threshold of *p* < 0.05 (two‐tailed, FWER‐corrected), and a fixed random seed of 42 for reproducibility.

The axis‐specific thickness and curvature were analyzed between groups using a two‐sample *t*‐test. Benjamini–Hochberg False discovery rate (FDR) correction was applied, and analyses that did not survive the correction were presented for exploratory visualization of hippocampal geometry but are not interpreted as confirmatory findings. Results were visualized using Matplotlib^36^ for 2D statistical maps and PyVista^37^ for 3D surface visualization.

Hedges' g effect sizes with 95% confidence intervals were calculated for all comparisons. All utilized software versions, reproducibility parameters and python packages are described at our publicly available repository at https://github.com/cherscofield/PART‐hippocampal‐morphometry‐KAJ‐lab.

## 3 RESULTS

### 3.1 Demographics and neuropathological characteristics

Demographics of the three groups—PART (TDP−), PART (TDP+), and YHIs—are shown in Table 1. Female sex represented 36% of the cohort, with no significant differences across groups. Age at MRI differed across the three groups, with YHIs being substantially younger than both PART groups. However, no difference in age at MRI was observed between PART(TDP−) and PART(TDP+) groups, confirming age‐matching for our primary comparison. Age at death did not differ between PART subgroups. The interval between MRI acquisition and mortality was 4 ± 3 years, with no significant differences observed among the PART groups. Additionally, the obtained at the last clinical visit prior to death did not vary between the PART subgroups. Furthermore, there were no differences in apolipoprotein E (*APOE*) ε4 carrier status among the PART subgroups.

**TABLE 1:** Demographic features of the PART subgroups and the young healthy adults’ group.

Parameter	PART (TDP−) *n* = 47	PART (TDP+) *n* = 19	YHIs *n* = 14	*p*‐value
Female sex	15 (32%)	7 (37%)	7 (50%)	0.47
Age at MRI (years)	83 (±7)	85 (± 5)	43 (±2)	<0.0001 (0.44*)
Age at death (years)	87 (±8)	89 (± 5)	—	0.43
MRI‐to‐death interval (years)	4 (±3)	4 (± 3)	—	0.74
MMSE score nearest death (max = 30)	25 (±4)	24 (± 4)	—	0.29
**Braak Stage**				0.36
1	4 (9%)	2 (11%)	—	
2	18 (38%)	3 (16%)	—	
3	17 (36%)	9 (47%)	—	
4	8 (17%)	5 (26%)	—	
^a^ **CERAD score**				0.002
0	13 (28.9%)	14 (73.7%)	—	
1	13 (28.9%)	4 (21.1%)	—	
2	19 (42.2%)	1 (5.3%)	—	
**Thal phase**				<0.0001
0	10 (21%)	13 (68%)	—	
1	13 (28%)	5 (26%)	—	
2	24 (51%)	1 (5%)	—	
*APOE* ε4 carrier	8 (17%)	2 (11%)		0.71

*Note*: The table presents the mean ± standard deviation (SD) for continuous variables and the count (percentage) for categorical variables. Comparisons across all three groups were analyzed using the Kruskal–Wallis test for continuous variables and chi‐square test for categorical variables. The Mann–Whitney U test was used for pairwise comparisons between PART (TDP−) and PART (TDP+) groups for continuous variables, and Fisher's exact test for categorical variables with small expected cell counts. The asterisk (*) indicates the *p*‐value derived from the Mann–Whitney U test comparing PART(TDP−) and PART(TDP+).

Abbreviations: *APOE*, apolipoprotein E; CERAD,  Consortium to Establish a Registry for Alzheimer's Disease; MMSE, Mini‐Mental State Examination; MRI, magnetic resonance imaging; PART, primary age‐related tauopathy; TDP, TAR DNA‐binding protein 43; YHIs, young healthy individuals.

^a^ CERAD data available for 45/47 PART(TDP−) cases.

Amyloid pathology burden differed significantly between PART subgroups. The PART (TDP+) group was enriched for Thal phase 0 and CERAD neuritic plaque score 0, while PART (TDP−) had a higher frequency of Thal 1–2 and CERAD 1–2. This inverse relationship between TDP‐43 positivity and amyloid plaque burden is consistent with prior evidence that TDP‐43 may inhibit early stages of Aβ fibrillization.

To address potential Aβ confounding, we performed sensitivity analyses restricted to cases with minimal amyloid pathology. First, analysis of Thal 0 cases only (*n* = 10 TDP−, *n* = 13 TDP+) showed well‐matched groups for age at MRI (87.0 ± 6.6 vs. 84.3 ± 4.8 years, *p* = 0.336) and sex (50% vs. 38.5% female, *p* = 0.685). Braak stage distributions spanned I–IV in both groups, with PART(TDP+) showing a slightly higher proportion of Braak III–IV (77%) compared to PART(TDP−) (40%), though this difference was not statistically significant. Volumetric comparisons showed consistent directional trends favoring smaller volumes in PART(TDP+) across all subfields; however, none reached statistical significance: left CA1‐body (4.1% reduction, *p* = 0.828), left subiculum‐body (6.0% reduction, *p* = 0.828), right CA1‐body (8.9% reduction, *p* = 0.163), and left whole hippocampus (5.1% reduction, *p* = 0.278). Second, analysis of CERAD 0 cases (*n* = 13 TDP−, *n* = 14 TDP+) yielded a Braak I‐homogeneous subset, matched for age (87.3 ± 5.8 vs. 84.6 ± 4.7 years, *p* = 0.216) and sex (46% vs. 36% female, *p* = 0.873). In this subset, right CA1‐body reached statistical significance (132.6 ± 19.3 vs. 116.5 ± 20.2 mm^3^, *p* = 0.044, 12.2% reduction), while left whole hippocampus showed a trend (3029.6 ± 369.6 vs. 2812.9 ± 381.8 mm^3^, *p* = 0.094, 7.2% reduction). The loss of statistical significance in the Thal 0 subset is primarily attributable to reduced statistical power (*n* = 23 vs. *n* = 66), rather than elimination of the TDP‐43 effect. The preservation of effect directions and magnitudes across both sensitivity analyses, with the CERAD 0/Braak I subset eliminating both amyloid and tau confounding, supports the interpretation that our primary findings reflect TDP‐43‐specific effects.

### 3.2 Volumetric analysis

Hippocampal subfield volumes were larger in the YHIs group for all left and right subfields compared to both PART groups. However, given the substantial age difference between YHIs and PART groups, these comparisons serve as a contextual reference for the magnitude of hippocampal abnormalities rather than age‐adjusted inferences.

In age‐matched comparisons between PART subgroups, the left CA1‐body subfield volume was significantly smaller in PART(TDP+) compared to PART(TDP−), representing a medium effect size (Table 2). Other subfield comparisons did not reach statistical significance after intracranial volume (ICV) normalization. To address potential confounding by differential Thal phase distribution, we performed ANCOVA controlling for Thal phase, Braak stage, age, and sex. After covariate adjustment, right CA1 body (*F* = 8.18, *p* = 0.006, partial *η* ^2^ = 0.127), left whole hippocampus (*F* = 5.50, *p* = 0.023, partial *η* ^2^ = 0.089), and left subiculum body (*F* = 4.22, *p* = 0.045, partial *η* ^2^ = 0.070) remained significantly smaller in PART (TDP+), demonstrating that TDP‐43 effects persist after accounting for amyloid and tau burden differences.

**TABLE 2:** Comparative analysis of hippocampal subfield volumes.

Hippocampal subfields	PART (TDP−) *n* = 47	PART (TDP+) *n* = 19	YHIs *n* = 14	*p*‐Value	g [95% CI]^b^
Left					
Presubiculum	1.5 × 10^−4^ ^b^	1.5 × 10^−4^ ^b^	1.9 × 10^−4^	0.0001 (0.77^a^)	0.11 [−0.42, 0.64]
Subiculum	2.2 × 10^−4^ ^b^	2.2 × 10^−4^ ^b^	2.8 × 10^−4^	0.0001 (0.19^a^)	0.26 [−0.28, 0.79]
CA1	3.6 × 10^−4^ ^b^	3.4 × 10^−4^ ^b^	4.4 × 10^−4^	0.0001 (0.048^a^)	0.48 [−0.06, 1.02]
CA2/3	1.3 × 10^−4^ ^b^	1.2 × 10^−4^ ^b^	1.5 × 10^−4^	0.009 (0.35^a^)	0.25 [−0.29, 0.78]
Right					
Presubiculum	1.4 × 10^−4^ ^b^	1.5 × 10^−4^ ^b^	1.9 × 10^−4^	0.0001 (0.19^a^)	−0.36 [−0.90, 0.18]
Subiculum	2.2 × 10^−4^ ^b^	2.2 × 10^−4^ ^b^	2.8 × 10^−4^	0.0001 (0.92^a^)	0.02 [−0.51, 0.55]
CA1	3.7 × 10^−4^ ^b^	3.6 × 10^−4^ ^b^	4.4 × 10^−4^	0.0001 (0.98^a^)	0.11 [−0.43, 0.64]
CA2/3	1.4 × 10^−4,^ ^b^	1.3 × 10^−4,^ ^b^	1.5 × 10^−4^	0.052 (0.95^a^)	0.18 [−0.35, 0.71]

*Note*: The table provides a comparative analysis of hippocampal subfield volumes across groups using the Kruskal–Wallis test. Volumes are presented as unitless values calculated by dividing subfields’ body volume by the estimated total intracranial volume (eTIV).

Abbreviations: CA, cornu ammonis; CI, confidence interval; PART, primary age‐related tauopathy; TDP, TAR DNA‐binding protein 43; YHIs, young healthy individuals.

^a^ The value in parentheses denotes the *p*‐value from the Mann–Whitney U test comparing PART (TDP−) versus PART (TDP+).

^b^ g = Hedges' g effect size with 95% confidence interval for the comparison between PART (TDP−) and PART (TDP+). Positive values indicate larger volumes in TDP−. Effect size interpretation: small (0.2), medium (0.5), large (0.8).

### 3.3 Correlation analysis

Spearman correlations between hippocampal morphometry and neuropathological staging revealed dissociable patterns across PART cases. Hippocampal thickness and Gaussian curvature showed no significant correlation with each other (left: *r* = −0.19, *p* = 0.12; right: *r* = −0.19, *p* = 0.13), confirming these represent independent morphometric measures. Neither thickness nor curvature significantly correlated with Braak NFT stage across all PART cases or within PART(TDP−) cases (all *p* > 0.3). However, in PART(TDP+) cases, right hippocampal Gaussian curvature showed a significant positive correlation with Braak stage (*ρ* = 0.512, *p* = 0.025), while thickness did not reach significance (*ρ* = −0.34, *p* = 0.16). Left hippocampal thickness showed a significant positive correlation with Thal amyloid phase across all PART cases (*ρ* = 0.309, *p* = 0.012), an association also observed in PART(TDP−) cases (*ρ* = 0.288, *p* = 0.050), while curvature showed no association with Thal phase. These findings suggest that thickness and curvature capture distinct aspects of hippocampal pathology, with curvature being particularly sensitive to tau burden in the context of TDP‐43 co‐pathology.

### 3.4 Geometry‐based thickness analysis

**FIGURE 1:**
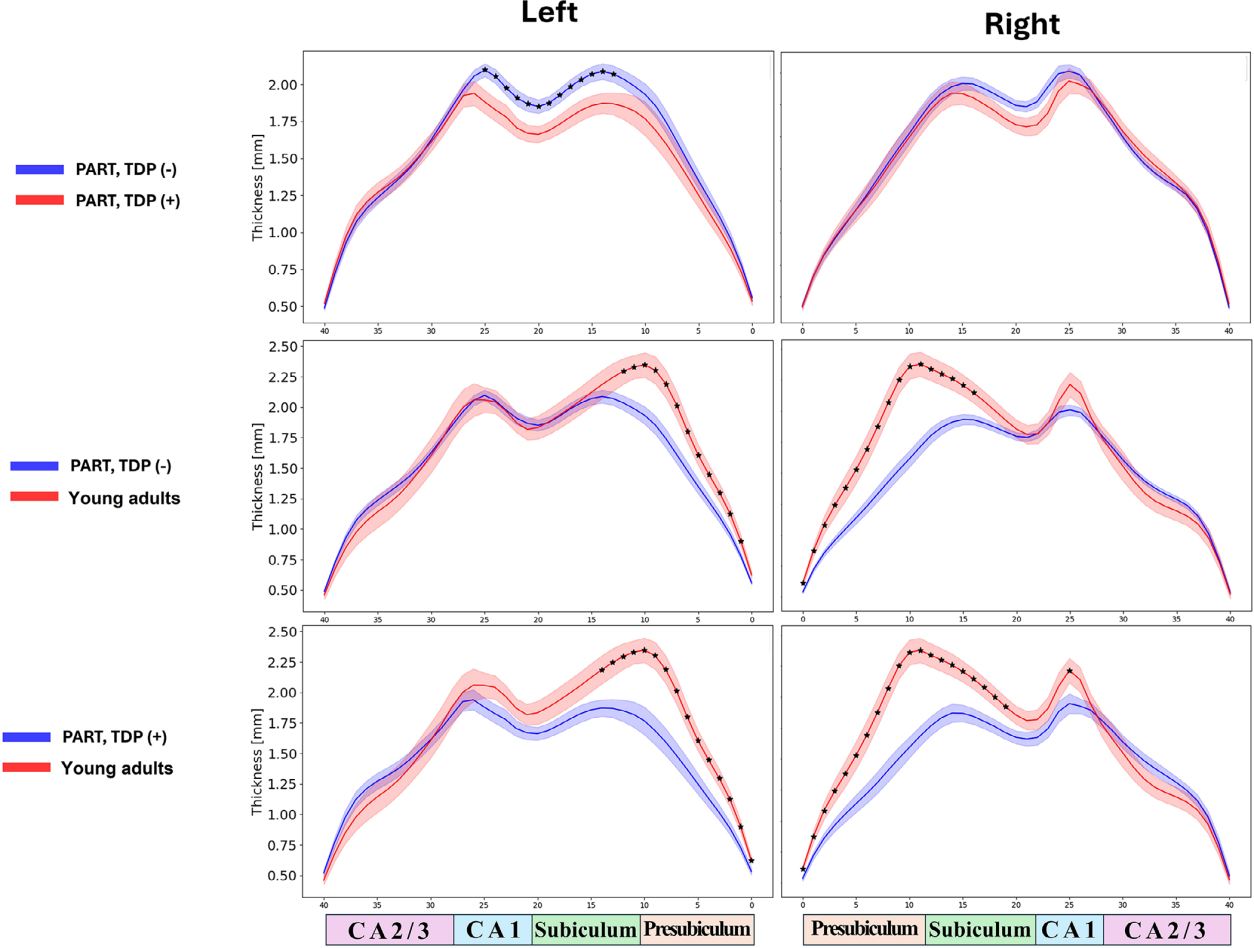
X‐Axis–wise hippocampal thickness comparison across study groups. The figure illustrates a comparison of hippocampal thickness along the x‐axis among the three study groups utilizing two‐sample *t*‐test. The labels for the groups under comparison are positioned on the left side of the figure, while the anatomical correspondence is indicated along the bottom. X‐axis shows subfields' thickness (mm). Results are presented for exploratory visualization as they did not survive FDR correction for multiple comparisons. The asterisk (*) indicates a significant difference between the plotted groups in the corresponding subfields. FDR, false discovery rate; Presub, presubiculum.

Axis‐specific *t*‐test analysis of hippocampal thickness showed the left subiculum and CA1 were significantly thinner in PART(TDP+) relative to PART(TDP−), with a similar trend observed on the right side (Figure 1). Notably, significant thinning of the presubiculum and subiculum in both PART(TDP−) and PART(TDP+) groups when compared to YHIs for both left and right hippocampi. These axis‐specific results are presented for exploratory visualization, as they did not survive FDR correction for multiple comparisons.

**FIGURE 2:**
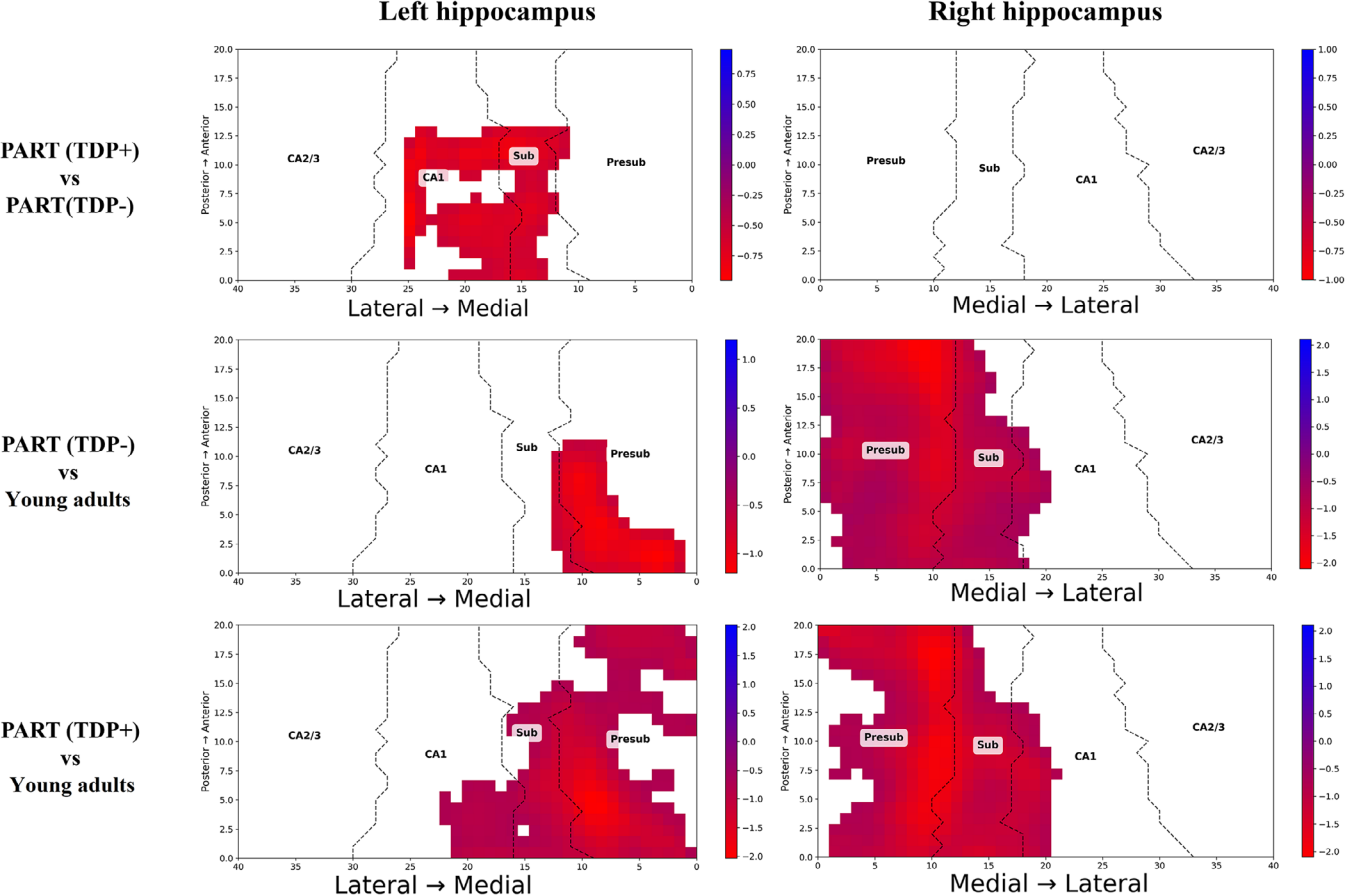
Point‐wise spatial comparative analysis of hippocampal thickness. The figure illustrates a comparative analysis of hippocampal thickness across the spatial axial plane of x and y coordinates of the hippocampus among the three study groups. The labels identifying the groups under comparison are positioned on the left side of the figure, while the anatomical correspondences are indicated along the bottom. Highlighted regions denote areas within the hippocampus that demonstrate significant differences in thickness between the groups being compared. A cluster‐based permutation test (5000 permutations, *p* < 0.05, FWER‐corrected) was employed to control for multiple comparisons while preserving spatial relationships. Effect sizes were calculated using Hedges' g. FWER, family‐wise error rate; Presub, presubiculum.

**FIGURE 3:**
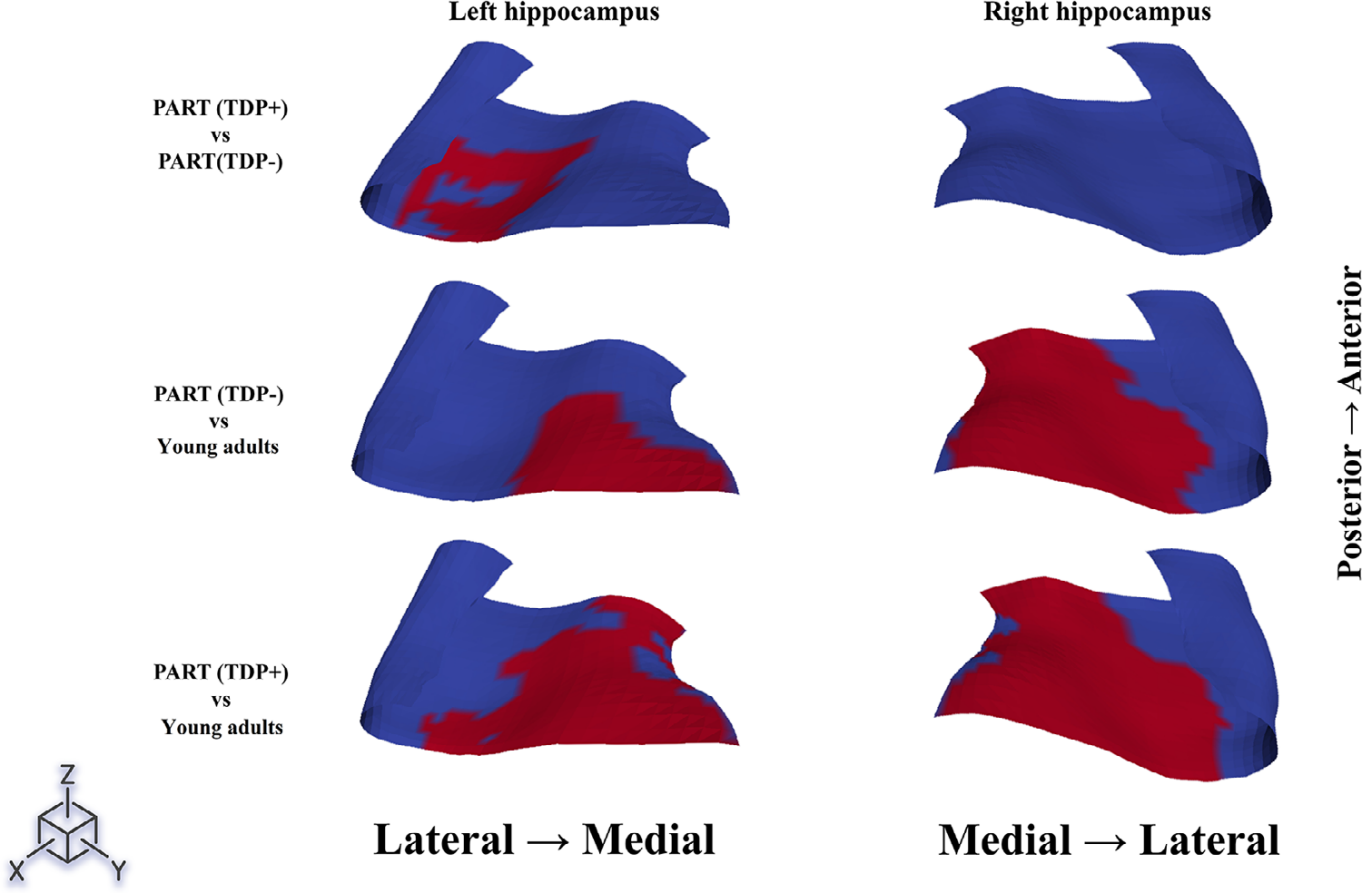
Affected thickness across the hippocampus among different groups. The figure presents the notable variations in thickness across the examined areas within the compared groups. The red markings across each hippocampus indicate the areas where significant differences in thickness exist between the groups being compared, as illustrated on the left side of the figure. Significance was determined using cluster‐based permutation testing (5000 permutations, *p* < 0.05, FWER‐corrected). FWER, family‐wise error rate.

Point‐wise cluster‐based permutation tests showed consistent results (Figure 2). The PART (TDP+) group had thinner left subiculum and CA1 subfields at the posterior end when compared to PART(TDP−). The areas of significant difference in thickness across the groups are shown in Figure 3. Presubiculum and medial subiculum were significantly thinner in both PART(TDP−) and PART(TDP+) relative to the YHIs group. The right hippocampus showed more extensive thinning across the presubiculum and subiculum compared to YHIs.

### 3.5 Geometry‐based curvature analysis

**FIGURE 4:**
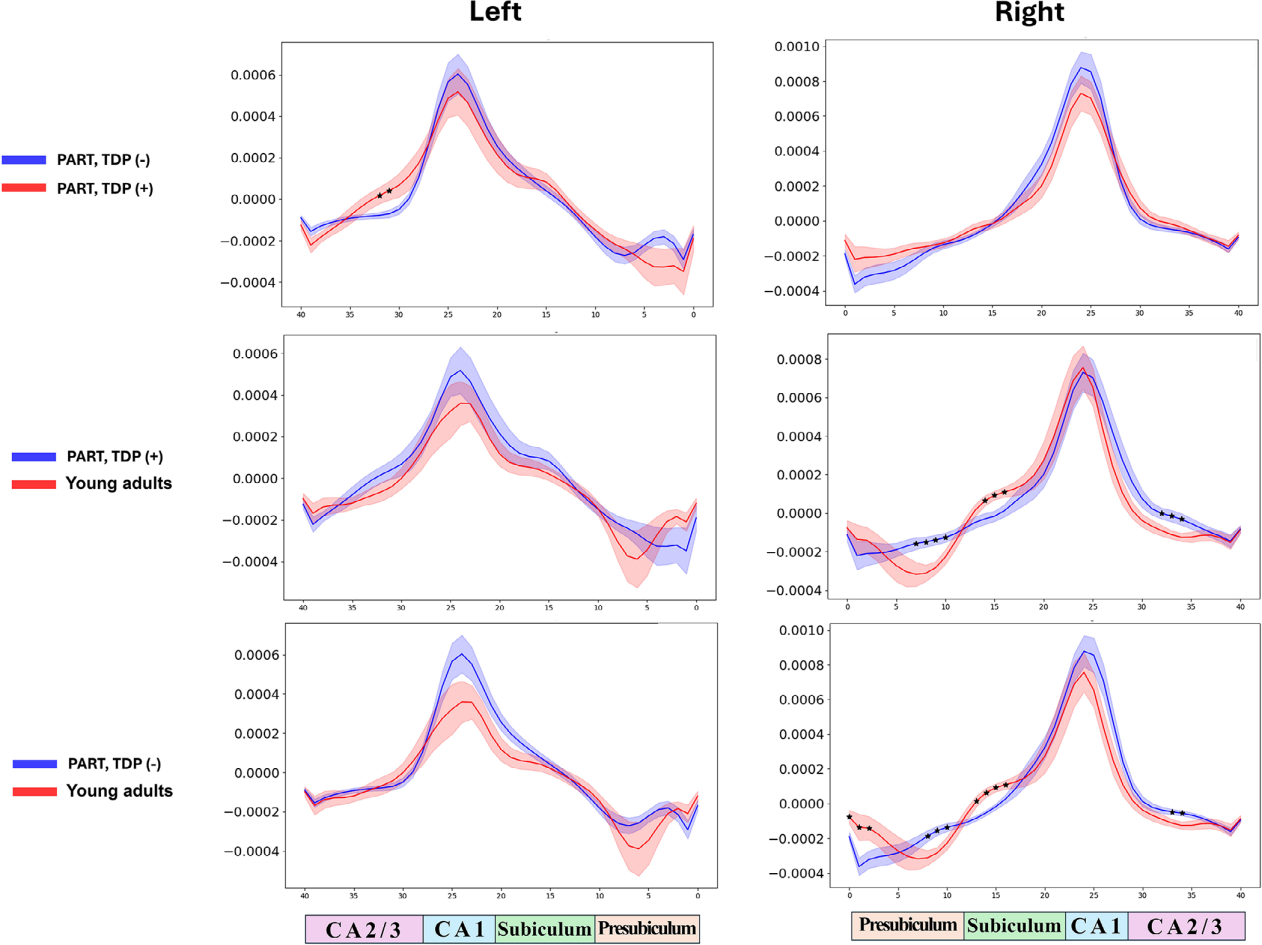
X‐axis‐wise hippocampal Gaussian curvature comparison across study groups. The figure illustrates a comparative analysis of hippocampal Gaussian curvature along the x‐axis across the three study groups. The group labels are positioned on the left side of the figure, while the anatomical correspondence is indicated along the bottom. X‐axis shows the subfields' Gaussian curvature (1/mm^2^). Two‐sample *t*‐test was employed for the comparative analysis. Results are presented for exploratory visualization as they did not survive FDR correction for multiple comparisons. The asterisk (*) indicates a significant difference between the plotted groups in the corresponding subfields. Presub, presubiculum.

Axis‐specific hippocampal Gaussian curvature was significantly different when comparing PART(TDP−) to PART(TDP+), with observed changes in curvature in the left CA2/3 subfield (Figure 4). The right side in the PART (TDP−) and PART (TDP+) groups compared to YHIs showed significant curvature changes across all subfields except for CA1. The left side showed some variability in curvature for both groups; however, it was not statistically significant.

**FIGURE 5:**
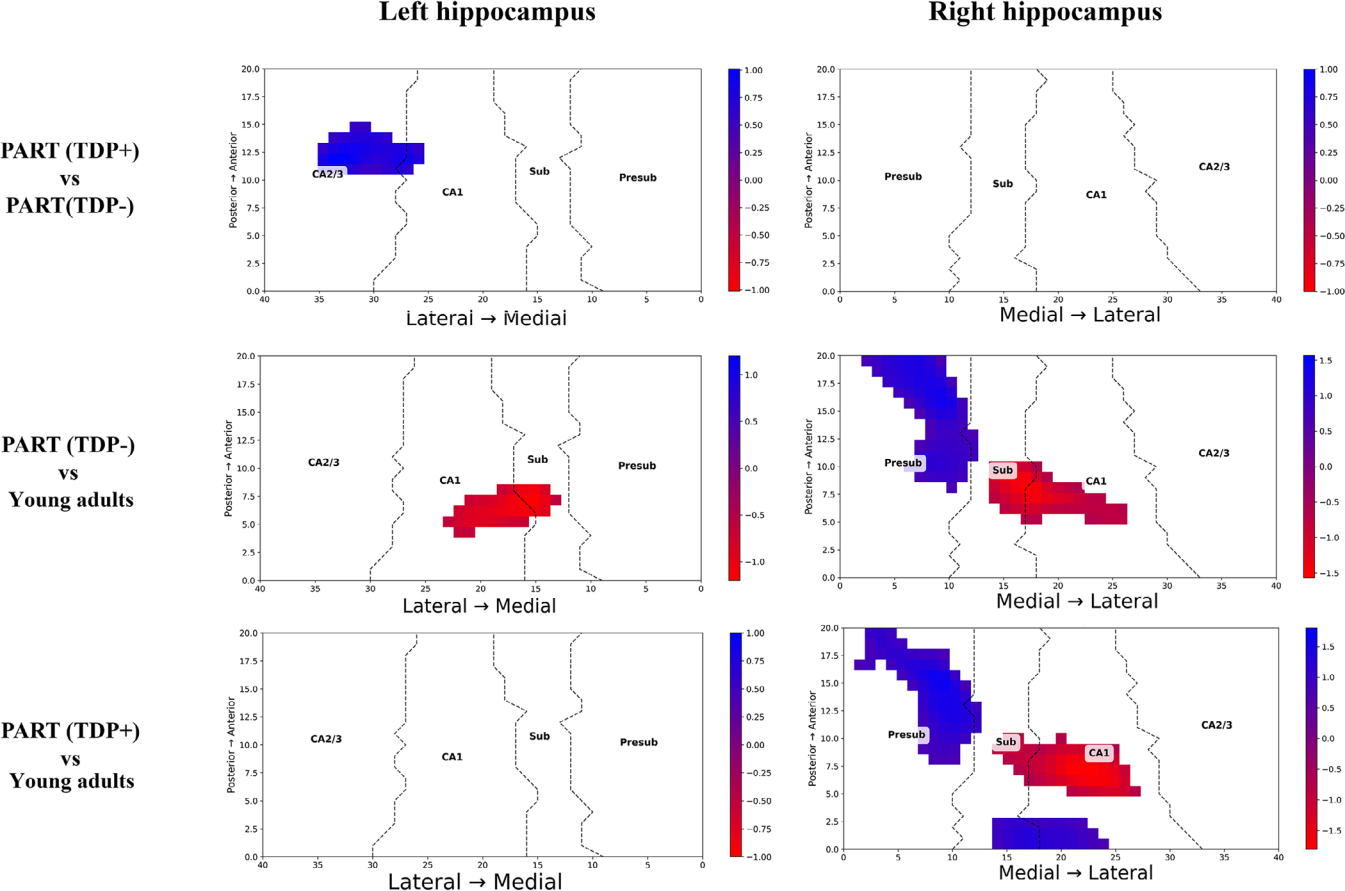
Point‐wise spatial comparative analysis of hippocampal Gaussian curvature. The figure presents a comparative analysis of the Gaussian curvature of the hippocampal surface across the spatial x and y coordinates of the hippocampus among the three study groups. The labels identifying the groups under comparison are located on the left side of the figure, while the anatomical correspondences are marked along the bottom. Highlighted regions indicate areas within the hippocampus that exhibit significant differences between the groups under comparison. The scale bar reflects the degree of Gaussian curvature change (1/mm^2^). A cluster‐based permutation test (5000 permutations, *p* < 0.05, FWER‐corrected) was utilized to control for multiple comparisons while preserving spatial relationships. Effect sizes (mean Hedges' g) for significant clusters: PART(TDP+) versus PART(TDP−)—left hemisphere g = −0.72; PART(TDP−) versus younger healthy individuals—left hemisphere g = −0.87, right hemisphere g = 0.17; PART(TDP+) versus younger healthy individuals—right hemisphere g = 0.11. FWER, family‐wise error rate; PART, primary age‐related tauopathy; Presub, presubiculum; TDP, transactive response DNA‐binding protein.

**FIGURE 6:**
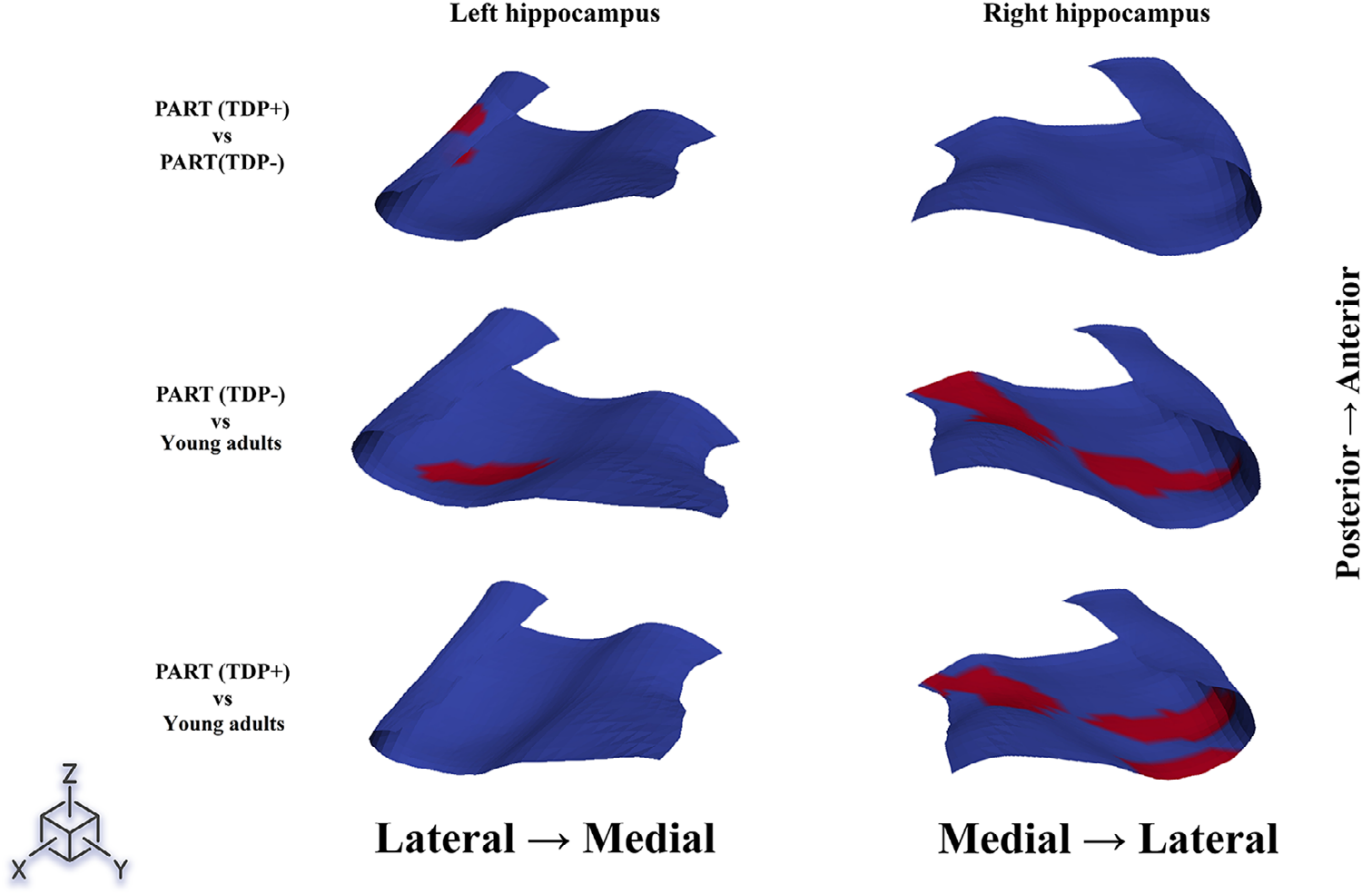
Affected curvature across the hippocampus among different groups. The figure illustrates the significant variations in Gaussian curvature observed across the examined regions within the groups compared. The red markings on each hippocampus highlight the areas where notable differences in curvature are present between the groups being analyzed, as depicted on the left side of the figure. Significance was determined using cluster‐based permutation testing (5000 permutations, *p* < 0.05, FWER‐corrected). FWER, family‐wise error rate.

Cluster‐based permutation tests, when comparing PART(TDP+) group to PART(TDP−), showed changes in curvature in the left lateral CA1 and CA2/3 subfield (Figure 5). Notably, CA2/3 curvature changes in TDP+ relative to TDP− occurred without corresponding thickness reduction in this region, suggesting that curvature deformation may be representing an earlier or complementary marker of TDP‐43‐associated structural changes. Additionally, significant variability in curvature of the posterior part of the left subiculum, right anterior presubiculum, right mid subiculum, and right mid‐to‐posterior CA1 when comparing the PART (TDP−) group to YHIs (Figures 5 and 6). The same effect was seen in the right hippocampus when comparing the PART(TDP+) group to YHIs, with more involvement of the posterior subiculum and CA1 subfields.

### 3.6 Hemispheric lateralization

Given the observed left‐predominant findings in PART(TDP+) and right‐predominant findings relative to YHIs, we performed formal laterality analyses. Laterality index analysis revealed no significant group differences in the direction of hemispheric asymmetry, indicating that volumetric left‐right balance was similar across groups. However, Asymmetry Index analysis, which quantifies the absolute magnitude of left‐right volume differences, revealed significant group differences. For subiculum‐body (*p* = 0.0134), PART(TDP+) showed substantially greater asymmetry (13.4%) compared to both PART(TDP−) (7.5%, post‐hoc *p* = 0.0096) and YHIs (6.9%), indicating that TDP‐43 pathology does not affect both hemispheres equally. For CA3‐body (*p* = 0.0227), YHIs showed greater natural asymmetry (17.4%) compared to PART(TDP−) (10.4%, post‐hoc *p* = 0.0101), with PART(TDP+) intermediate (11.4%). Presubiculum‐body and CA1‐body showed no significant AI differences. Additionally, mixed‐effects models revealed a significant group × hemisphere interaction for presubiculum‐body (*p* = 0.042), indicating that TDP‐43 effects were left‐predominant with larger effect sizes in the left hemisphere (Cohen's *d* = 0.53) compared to the right hemisphere (*d* = 0.10). Additionally, hemisphere‐specific analyses confirmed that TDP‐43‐associated volume reductions were significant in the left hemisphere for CA1‐body (*p* = 0.003, *d* = 0.85), subiculum‐body (*p* = 0.003, *d* = 0.85), and whole hippocampus (*p* = 0.003, *d* = 0.83), while right hemisphere effects were smaller and generally non‐significant: CA1‐body (*p* = 0.013, *d* = 0.70), subiculum‐body (*p* = 0.085, *d* = 0.48), and whole hippocampus (*p* = 0.070, *d* = 0.50).

## 4 DISCUSSION

Using a geometry‐based algorithm, we investigated hippocampal morphometry across three groups: PART(TDP−), PART(TDP+), and YHIs. PART(TDP+) was associated with thinning of the posterior‐lateral hippocampus, especially the subiculum and CA1, and curvature changes in CA1 and CA2/3 on the left side. In contrast, PART(TDP−) was associated with thinning and curvature changes of the medial hippocampus, particularly presubiculum and subiculum, with right‐side alterations being more pronounced.

Our findings indicate that PART without TDP‐43 is associated with thinning in presubiculum and subiculum subfields, as well as curvature deformation in these subfields and CA1, more pronounced on the right side. A multi‐modal autopsy‐MRI study similarly reported thinning in these subfields associated with tau pathology in cases negative for Aβ and TDP‐43.^38^ Other research indicated that tau NFTs appear earliest in the presubiculum/subiculum complex defining Braak I–II, with neuronal loss preceding CA1 degeneration, even in cases without TDP‐43.^39^, ^40^, ^41^, ^42^ This matches PART topography, where presubiculum and subiculum volumes decrease before the rest of the hippocampus.^43^, ^44^ Our findings suggest a subfield‐specific and hemispheric lateralized effect of PART in absent‐to‐low Aβ and negative TDP‐43.

A key neuropathological study found that 49% of PART cases demonstrated asymmetry in at least one hippocampal subregion, with 79% of asymmetric cases displaying CA2 subregion asymmetry, and 19% showed differences in Braak scores between hemispheres.^45^ Research in mild cognitive impairment (MCI) has shown that the right hippocampus demonstrates more pronounced atrophy compared to the left, with asymmetry indices increasing through the AD continuum.^46^, ^47^ A recent study revealed that tau asymmetry patterns are strongly associated with Aβ laterality patterns in AD, suggesting hemispheric vulnerability patterns may be established early and influence subsequent pathological development.^48^ Several factors may have contributed to the right‐side predominance in PART observed in our study, including normal hippocampal asymmetry,^49^, ^50^ differential vulnerability to aging,^51^ and distinct right hemisphere connectivity patterns.^52^, ^53^, ^54^

Our findings provide new insights into hippocampal morphometric deformation associated with TDP‐43 in PART. A recent study demonstrated that TDP‐43 was uniquely associated with inward deformation in bilateral CA1 and subiculum in AD patients.^19^ These findings correspond with our thickness results, as reduced thickness could be interpreted as inward surface deformation. Additionally, surface curvature deformation was seen in the lateral left CA1 extending to medial CA2/3, with the highest effect size in CA1. The recent study controlled for Aβ (minimal in our cohort) and PHF‐tau (limited to Braak I–IV and present in both groups). Importantly, our covariate‐adjusted ANCOVA demonstrated that TDP‐43 effects persist after accounting for Thal phase and Braak stage, with left subiculum‐body, left whole hippocampus, and right CA1‐body remaining significantly associated with TDP‐43 status. The discrepancy that our findings are confined to the left hippocampus may be attributed to differing methodologies in hemispheric fixation during autopsy.^19^ We previously reported that CA1 is a primary subfield associated with TDP‐43 pathology in PART,^15^ and the finding that left CA1 was significantly smaller in PART(TDP+) further demonstrates CA1 vulnerability to TDP‐43 pathology and suggests probable hemispheric specificity.

The observation that CA2/3 shows curvature changes without reduced thickness may support differential sensitivity of these morphometric measures, possibly suggesting curvature changes represent an earlier phenomenon prior to thickness reduction. Correlation analyses provided empirical support: hippocampal thickness and Gaussian curvature showed no significant correlation, confirming these are independent measures capturing distinct aspects of pathology. Notably, in PART(TDP+) cases, right hippocampal Gaussian curvature showed a significant positive correlation with Braak NFT stage while thickness did not, suggesting curvature may be particularly sensitive to tau burden in the context of TDP‐43 co‐pathology. Additionally, left hippocampal thickness showed a significant positive correlation with Thal amyloid phase across all PART cases, while curvature showed no association—further demonstrating the differential sensitivity of these measures to distinct proteinopathies. The statistical independence of thickness and curvature supports their complementary value in characterizing hippocampal degeneration. Surface curvature may serve as a more sensitive marker for neurodegeneration than thickness, detecting subtle alterations that precede volumetric changes.^16^, ^55^, ^56^ Curvature analysis has demonstrated superior statistical power compared to volume measurements, capturing fine shape variations missed by traditional analyses.^16^, ^55^, ^57^, ^58^ Biologically, thickness changes reflect neuronal loss and dendritic atrophy, while curvature changes may capture geometric deformations before substantial tissue loss. Therefore, surface curvature changes could reflect alterations in the local geometry of the hippocampal structure, which may occur before sufficient neuronal loss accumulates to produce measurable thickness reduction. Curvature can also serve as a proxy for internal hippocampal structure—the dentate gyrus and CA4 lie within the C‐shaped curve formed by CA1‐3 and subiculum, so internal volume loss would compress this curve, producing increased curvature in CA1.^17^, ^56^, ^57^

TDP‐43 in PART is associated with worse memory impairment and brain atrophy.^14^, ^59^ Episodic and autobiographical memory are associated with the left hippocampus, while the right is associated with spatial memory.^60^, ^61^ CA1 has been specifically implicated in episodic memory formation and verbal memory processes.^62^, ^63^ The selective vulnerability of left CA1 to TDP‐43 may help explain why PART with TDP‐43 is associated with worse cognitive impairment, particularly in memory domains^7^, ^22^, ^40^, ^41^, ^64^ and suggests a lateralized effect of TDP‐43 in PART.^2^, ^40^ Further investigation utilizing bilateral TDP‐43 staining is necessary to confirm this observation.

It is noteworthy that our PART(TDP+) cases exhibited a higher frequency of Thal 0 compared to PART(TDP−), contrary to the postulation that TDP‐43 promotes Aβ accumulation.^65^ TDP‐43, tau, and Aβ engage in a complex three‐way interaction.^66^ TDP‐43 inhibits Aβ fibrillization during early stages, resulting in less dense and shorter filaments, though it does not affect mature fibrils.^65^ This suggests TDP‐43 may impede conversion of soluble Aβ into fibrillar plaques, allowing PART brains with TDP‐43 to remain plaque‐free (Thal 0), whereas TDP‐43 absence permits plaques to emerge. Thus, TDP‐43 positivity and Thal‐detectable Aβ deposition may be biologically antagonistic; whether this is specific to PART requires further investigation.

Currently, TDP‐43 can only be diagnosed at autopsy, creating a significant diagnostic gap given that TDP‐43 co‐pathology occurs in 20%–50% of PART cases and is associated with accelerated cognitive decline.^66^ The distinct hippocampal morphometric signature we identified—characterized by left CA1/subiculum changes with large effect sizes—suggests that standard clinical MRI may provide a non‐invasive window into TDP‐43 status. Several features support translational potential: the analysis uses standard 3T T1‐weighted MRI widely available in clinical settings, the HIPSTA pipeline is fully automated, enabling scalable implementation, and large effect sizes indicate a robust signal that may translate to clinically useful diagnostic accuracy. Near‐term applications include enrichment strategies for TDP‐43‐targeted clinical trials, where hippocampal shape analysis could complement demographic and cognitive screening to identify likely TDP‐43 carriers. Full clinical implementation will require validation in larger independent cohorts, development of diagnostic algorithms with established sensitivity and specificity, and demonstration of clinical utility for prognosis and treatment selection.

This study represents a novel assessment of geometry‐based hippocampal subfield analysis to investigate TDP‐43 effects in PART. The pathological characteristics are well‐defined with a sufficient sample size. Regarding limitations, first, the YHIs group serves as an anatomical reference rather than a true comparison group, as age‐matched controls without tau pathology are not biologically feasible; our primary analyses focus on TDP+ versus TDP− comparisons. Second, clinical T1‐weighted MRI (1 mm^3^) provides insufficient internal contrast, and the histological foundations of geometry measurements remain speculative, necessitating ex vivo studies. Third, TDP‐43 staining was performed only on the left hemisphere; we cannot determine whether left‐sided changes reflect true lateralization or are confounded by unilateral sampling. Fourth, FreeSurfer combines CA2 and CA3 into a single label, because CA2 is anatomically narrow (∼1–2 mm) with a gradual cytoarchitectonic transition to CA3; our CA2/3 findings cannot determine whether changes are driven by CA2, CA3, or both. Future studies using 7T MRI, ex vivo imaging with histological correlation, and bilateral TDP‐43 assessment are essential. Until such data are available, our lateralization findings should be considered provisional and hypothesis‐generating.

In conclusion, we demonstrate different patterns of hippocampal subfield thinning and curvature changes associated with PART and TDP‐43. PART(TDP−) was associated with more extensive right hippocampal changes in presubiculum and subiculum, while PART(TDP+) was associated with left CA1 thinning and curvature deformation, followed by left subiculum thinning and CA2/3 curvature changes. These findings indicate potential distinct lateralization effects of TDP‐43 and PART requiring further investigation, and suggest that shape deformation techniques are sensitive to subtle hippocampal changes in PART.

## CONFLICT OF INTEREST STATEMENT

The authors have no conflicts of interest to declare. Author disclosures are available in the supporting information.

## CONSENT STATEMENT

All human subjects provided informed consent for inclusion in research studies.

## Supporting information



Supporting information

Supporting information

Supporting information

Supporting information
