# Supplementary material for: Hippocampal subfield thickness and shape analysis in examining the impact of TDP‐43 in primary age‐related tauopathy
Source: Alzheimers Dement. 2026 Mar 8;22(3):e71267. doi: 10.1002/alz.71267 (PMC12967478; doi:10.1002/alz.71267)
Supplement: Supplementary file 1 — Supporting information [file ALZ-22-e71267-s004.docx]

Supplementary Material

# Supplementary Table S1: MRI Acquisition Parameters for study cases

| Parameter | Value |
| --- | --- |
| Site | Mayo Clinic, Rochester, MN |
| Field Strength | 3.0 Tesla |
| Manufacturer | GE Medical Systems |
| Scanner Models | SIGNA HDx |
| Receive Coil | 8-channel head coil (8HRBRAIN) |
| Sequence | 3D Sagittal MP-RAGE |
| Repetition Time (TR) | 6.6-7.2 ms |
| Echo Time (TE) | 2.8-3.1 ms |
| Inversion Time (TI) | 900 ms |
| Flip Angle | 8 degrees |
| Acquisition Matrix | 256 x 256 |
| Voxel Size | 1.0 x 1.0 x 1.0 mm |
| Acquisition Time | ~5 min |
| Imaging Frequency | 127.7 MHz |
