# Supplementary material for: Hippocampal subfield thickness and shape analysis in examining the impact of TDP‐43 in primary age‐related tauopathy
Source: Alzheimers Dement. 2026 Mar 8;22(3):e71267. doi: 10.1002/alz.71267 (PMC12967478; doi:10.1002/alz.71267)
Supplement: Supplementary file 2 — Supporting information [file ALZ-22-e71267-s003.docx]

Supplementary Material

**Supplementary Table S2: MRI Quality Comparison
(Included vs Excluded PART Cases)**

| MRI Quality Grade | Included (n=66) | Excluded (n=7) |
| --- | --- | --- |
| Grade 2 | 58 (87.9%) | 6 (85.7%) |
| Grade 3 | 8 (12.1%) | 1 (14.3%) |
| Grade 1 | 0 (0%) | 0 (0%) |
| Grade 4 | 0 (0%) | 0 (0%) |

The Mayo Clinic MRI Quality Control (QC) uses a 4-level scale for grading the quality of structural MRI scans, including MPRAGE sequences. Grades 1-3: Deemed acceptable or usable for analysis. Grade 4: Considered a failure or unusable scan.
