# Supplementary material for: Hippocampal subfield thickness and shape analysis in examining the impact of TDP‐43 in primary age‐related tauopathy
Source: Alzheimers Dement. 2026 Mar 8;22(3):e71267. doi: 10.1002/alz.71267 (PMC12967478; doi:10.1002/alz.71267)
Supplement: Supplementary file 3 — Supporting information [file ALZ-22-e71267-s001.docx]

Supplementary Material

**Methods:**

*HIPSTA Algorithm pipeline*

Anterior and posterior hippocampal boundaries were defined at transitions between hippocampal body and head/tail based on FreeSurfer segmentation labels; the hippocampal head was excluded because it folds laterally onto itself preventing reliable unfolding, and the tail was excluded due to insufficient anatomical detail at clinical resolution. Medial and lateral boundaries were automatically identified using the anisotropic Laplace-Beltrami operator with Neumann boundary conditions, where the zero level sets of the first eigenfunction attract to high-curvature zones, localizing the medial boundary at the presubiculum/entorhinal cortex junction and the lateral boundary at the CA3/CA4 junction.

Three Laplace equations were solved via finite element method (FEM) to create a harmonic mapping defining an intrinsic 3D coordinate system: u(x,y,z) representing the medial-to-lateral dimension, v(x,y,z) representing the posterior-to-anterior dimension, and w(x,y,z) representing the interior-to-exterior dimension. An approximate mid-surface (w=0.5 level set) was extracted, and a regular rectangular grid (41 × 21 = 861 vertices per hemisphere, approximately 1–2 mm spacing) was prescribed on this surface. Local thickness at each grid point (uᵢ, vⱼ) was computed by tracing streamlines along the interior-exterior dimension using Thickness(uᵢ, vⱼ) = ∫[w=0 to w=1] ||∇w|| dw, where ||∇w|| is the magnitude of the gradient of the interior-exterior parametric function; thickness values are expressed in millimeters and measured perpendicular to the mid-surface, corresponding to the radial orientation from hippocampal core to external boundary.

Gaussian curvature (K) was computed at each mid-surface vertex as K = κ₁ × κ₂, where κ₁ and κ₂ are the principal curvatures derived from the triangulated mid-surface using standard differential geometry: surface normal vectors computed at each vertex from adjacent triangles, curvature tensor computed from local surface normal variations, eigenvalue decomposition of the curvature tensor yielding κ₁ and κ₂, and Gaussian curvature computed at each vertex; Gaussian curvature is expressed in units of 1/mm², where K > 0 indicates convex surface geometry, K < 0 indicates saddle/concave geometry, and K = 0 indicates flat or cylindrical geometry. The point-wise analysis covered the hippocampal body including presubiculum, subiculum, CA1, and CA2/3 (combined due to FreeSurfer segmentation limitations). A curvature-based spatial alignment procedure was applied to correct for individual differences in hippocampal extent along the medial-lateral axis, ensuring anatomically consistent correspondence across subjects. No explicit spatial smoothing kernel was applied to thickness or curvature measurements; implicit regularization was provided by mild Laplacian mesh smoothing, inherent smoothness of Laplace equation solutions, and FreeSurfer segmentation spatial priors, while the cluster-based permutation testing approach inherently accounts for spatial correlation without requiring explicit pre-smoothing.
